# Current nursing and midwifery contribution to leading digital health policy and practice: An integrative review

**DOI:** 10.1111/jan.16265

**Published:** 2024-06-30

**Authors:** Gillian Janes, Lorna Chesterton, Vanessa Heaslip, Joanne Reid, Bente Lüdemann, João Gentil, Rolf‐André Oxholm, Clayton Hamilton, Natasha Phillips, Michael Shannon

**Affiliations:** ^1^ School of Nursing Anglia Ruskin University Cambridge UK; ^2^ Centre for Health Systems and Safety Research Macquarie University Macquarie Park New South Wales Australia; ^3^ Faculty of Health and Education Manchester Metropolitan University Manchester UK; ^4^ School of Health and Society University of Salford Manchester UK; ^5^ Department of Social Sciences University of Stavanger Stavanger Norway; ^6^ School of Nursing and Midwifery Queen's University Belfast UK; ^7^ Norwegian Nurses Organisation Oslo Norway; ^8^ Public Health Unit Baixo Mondego Portugal; ^9^ Regional Office for Europe, World Health Organization Copenhagen Denmark; ^10^ Chief Nurse's Office, NHS England London UK; ^11^ Faculty of Nursing and Midwifery, Royal College of Surgeons in Ireland Dublin Ireland

**Keywords:** digital health, health policy, health workforce, integrative review, leadership, midwives, nurses

## Abstract

**Aim:**

To review the current nursing and midwifery contribution to leading digital health (DH) policy and practice and what facilitates and/or challenges this.

**Design:**

Integrative literature review.

**Methods:**

Pre‐defined inclusion criteria were used. Study selection and quality assessment using the appropriate critical appraisal tools were undertaken by two authors, followed by narrative synthesis.

**Data Sources:**

Six databases and hand searching for papers published from 2012 to February 2024.

**Findings:**

Four themes were identified from 24 included papers. These are discussed according to the World Health Organization's Global Strategic Directions for Nursing and Midwifery and indicate nurses/midwives are leading DH policy and practice, but this is not widespread or systematically enabled.

**Conclusion:**

Nurses and midwives are ideally placed to help improve health outcomes through digital healthcare transformation, but their policy leadership potential is underused.

**Implications for the profession and/or patient care:**

Nurses/midwives' DH leadership must be optimized to realize maximum benefit from digital transformation. A robust infrastructure enabling nursing/midwifery DH policy leadership is urgently needed.

**Impact:**

This study addresses the lack of nursing/midwifery voice in international DH policy leadership. It offers nurses/midwives and health policymakers internationally opportunity to: drive better understanding of nursing/midwifery leadership in a DH policy context; enhance population outcomes by optimizing their contribution; Develop a robust infrastructure to enable this.

**Reporting Method:**

Reporting adheres to the EQUATOR network, Preferred Reporting Items for Systematic Review and Meta‐Analysis (PRISMA) guidelines.

**Patient or Public Contribution:**

No patient or public contribution.

**What does this paper contribute to the wider global clinical community?**

- Robust evidence synthesis of the nursing/midwifery contribution to digital health policy leadership.
- Evidences the beneftis of optimising the nursing/midwifery contribution.
- Evidence‐based recommendations for enabling a greater nursing/midwifery digital health policy leadership contribution.

## 1 INTRODUCTION

The topic of digital health (DH) is of international relevance as digital technology has revolutionized delivery of healthcare services across the world, bringing the potential to improve efficiency, patient safety and health outcomes and influence workforce planning (ICN, 2023). If implemented from an equity perspective, digital technology has the potential to address global health inequality by improving access to professionals through telemedicine and improved data analytics (United Nations, 2016), supporting the achievement of the United Nations (UN) sustainable development goals (SDGs; UN, 2015).

## 2 BACKGROUND

Digital health may be defined as:

‘The field of knowledge and practice associated with the development and use of digital technologies to improve health…expands the concept of eHealth to include digital consumers, with a wider range of smart devices and connected equipment’. (WHO, 2021a, p. 11).

As this definition indicates, DH encompasses the digital technologies that enable DH practices and functions such as remote consultation, smartphone apps and wearable technology for physiological health measurement. Existing literature on digital technology, however, is very broad (Krick et al., 2019) without the term always being clearly defined; current applications are varied, including for example, remote delivery of care for people with Parkinson's disease (Tamplin et al., 2023); and cardiac rehabilitation and secondary prevention programmes (de Moel‐Mandel et al., 2023). The term DH is therefore used throughout this paper to represent areas that are commonly considered part of this concept, that is, artificial intelligence, big data, blockchain, health data, health information systems, infodemic, the Internet of Things and telemedicine (WHO Europe, 2022).

Arguably, a positive legacy of COVID‐19 has been the global acceleration of DH, which has impacted all sectors, facilitating innovative service delivery (Agnew, 2022; Brommeyer & Liang, 2022). Many of the solutions for addressing workforce shortages, finite resources and the challenges of caring for increasing numbers of older people lie within the arena of DH (Webster, 2020). Strategic goals of the European Union (EU) and World Health Organization (WHO; WHO Europe, 2022) advocate DH as a way of improving the quality and efficiency of healthcare, by enabling new care delivery methods and empowering citizens and communities (Ahonen et al., 2016; de Raeve et al., 2017). Such flexibility means that patients, supported by nurses, can use DH for remote monitoring; teleconsultations (de Raeve et al., 2017), information access and mobile applications for care and health promotion (Honey & Westbrooke, 2016); improving disease prevention, health outcomes and quality of life (de Raeve et al., 2017; Honey & Westbrooke, 2016). However, this needs to be within international guidelines designed to enable policymakers and key stakeholders to make informed decisions regarding appropriate investment in and implementation of DH, which are still evolving (WHO, 2019).

Nursing has a pivotal role in healthcare delivery, so must adapt to new ways of working by acquiring digital skills that optimize practice and contribute to the SDGs (WHO, 2021a). However, the DH discourse often positions nurses as disengaged from technology (Agnew, 2022; Sadoughi et al., 2016) with healthcare services lagging behind because of historic underfunding in information technology (IT) infrastructure (Hutchings, 2020) and disparate, fragmented, systems that lack interoperability (Ariosto et al., 2018; de Raeve et al., 2017; Honey & Westbrooke, 2016; Hussey et al., 2015, 2017, 2021; ONMSD, 2022; Remus, 2016a; Remus & Donelle, 2019). These challenges are globally recognized and exacerbated in low‐ and lower‐middle‐income countries (WHO, 2021a). Furthermore, nurse education is not effectively preparing nurses for new digitally enabled ways of delivering healthcare (Morris et al., 2023); although this is not unique to nursing, as illustrated by calls for competency‐based DH education for all healthcare professions (Nazeha et al., 2020; Topol, 2019). These challenges are highlighted in the WHO Global Strategic Directions for Nursing and Midwifery (WHO, 2021b), and as the key global strategic nursing and midwifery policy, this framework was used to frame our discussion.

International organizations acknowledge the important role of nurses in DH development, yet their contribution to national and international strategy development is often not visible (Sadoughi et al., 2016). More staff are using telehealth applications to deliver care and access data (Skiba, 2017) leading to national developments like *‘*Every nurse an e‐nurse’ (RCN, 2018) in the United Kingdom. Despite this, the contribution of nurses and midwives to the strategic development of digital healthcare is poorly defined and little is known about the role and impact of leaders in the profession (Troncoso & Breads, 2021). Research is therefore needed regarding digitally enabled nursing practice and using data held in digital systems to inform policy (O'Connor et al., 2024).

The adoption of the Digital Action Plan by the WHO Regional Committee for Europe provides the potential opportunity to develop a platform for a joint policy brief (International Council of Nurses [ICN]/WHO) regarding the contribution of the nursing and midwifery profession to DH and what is needed to enable this; this literature review aimed to provide a review of the evidence base and recommendations to underpin such policy development.

## 3 THE REVIEW

### 3.1 Aim

To synthesize and summarize the literature regarding the leadership contribution of nursing and midwifery to DH policy and practice by addressing the following questions:

1. What is the current nursing and midwifery contribution to leading DH policy and practice?
2. What facilitates and/or constrains this contribution?

### 3.2 Design

An integrative review was chosen to enable the inclusion of diverse information (theoretical, conceptual, policy and empirical) (Souza et al., 2010) which together enable better understanding of the phenomenon (Godin et al., 2015) for policy development (Grant & Booth, 2009). The recommended six phases were followed, namely: preparing the guiding question; searching the literature; data collection; critical analysis of the studies; discussion of the findings; and presentation of the review (Souza et al., 2010). The protocol was pre‐registered at PROSPERO (york.ac.uk), ID: CRD42023420369, and the review followed the Preferred Reporting Items for Systematic Review and Meta‐Analysis (PRISMA) guidelines (Page et al., 2021).

### 3.3 Search methods

Systematic searching was supported by a specialist academic librarian and included a test search for appropriate scope and sensitivity. Six databases (PubMed, Web of Science, CINAHL, Cochrane Database of Systematic Reviews, OpenGrey and Ethos (British Library e‐theses online)) were searched for papers published from 2012 to February 2024 using:

Nurs* OR Midwi* AND "digital health" OR *e*‐health OR telehealth OR "mobile health" OR mhealth OR tele‐health OR ehealth AND policy OR strateg*

Searching relevant websites, including WHO; European Commission; ICN; nursing/midwifery professional bodies; key DH reviews (Topol, 2019); and specialist journals (Nursing Informatics, NI), plus citation tracking/reference list searching of included papers complemented database searching.

### 3.4 Inclusion/exclusion criteria

The search was limited to papers published in 2012 up to February 2024 when the final searches were undertaken. Document selection was determined using the inclusion and exclusion criteria outlined in Table 1 and focused on papers published in English that addressed registered nurses' or midwives' contribution to or leadership of DH policy and practice, in any nursing or midwifery context.

**TABLE 1:** Inclusion and exclusion criteria.

Inclusion criteria	Exclusion criteria
Registered nurses or midwives	Student nurses or midwives, healthcare assistants, other health and social care staff where we cannot extract data pertaining to nurses/midwives
Digital health leadership (policy and practice)	Not primarily focused on nursing or midwifery leadership of digital healthcare Focused solely on implementation of digital services/solutions in which nursing or midwifery leadership is not evident/reported
Focuses on nursing or midwifery leadership	Generic leadership pieces or focusing on leadership by other groups
Any nursing or midwifery context—primary, secondary and social care; local, regional, national and international level policy and practice	
Peer‐reviewed, empirical studies—any design, grey literature (including policy documents and expert opinion) PhD theses Book chapters	Blogs Conference abstracts
Published in English, Swedish, Portuguese or Danish	Other languages except where there is relevant translation expertise within the review team
Published from 2012 onwards	Published before 2012

### 3.5 Study selection process

**FIGURE 1:**
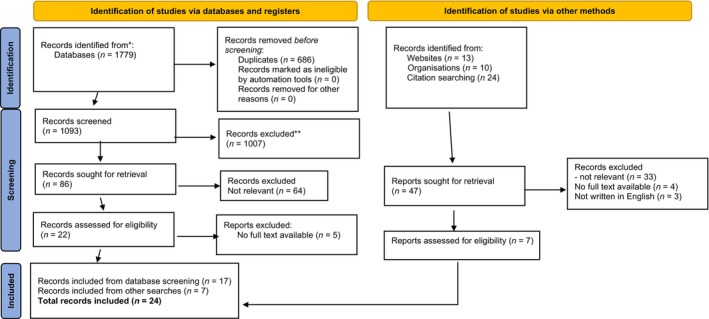
Screening and selection process. *Consider, if feasible to do so, reporting the number of records identified from each database or register searched (rather than the total number across all databases/registers). **If automation tools were used, indicate how many records were excluded by a human and how many were excluded by automation tools. Source: Page et al. (2021). For more information, visit: http://www.prisma‐statement.org/.

Database searches identified 1779 papers, which were exported to Endnote 20 and duplicates removed. A further 47 papers were identified through citation and hand searching. Figure 1 depicts the full screening and selection process. Title and abstract screening of all citations, followed by full‐text screening, was completed by one author using the pre‐defined eligibility criteria and 10% of exclusions and all ‘maybe’ papers were independently screened at each stage.

### 3.6 Quality appraisal

All included papers were independently assessed for methodological quality by author dyads using appropriate, validated tools: QuADS (Harrison, 2021) for methodologically diverse research studies; AACODS checklist (Tyndall, 2010) for grey literature or CASP (Critical Appraisal Skills Programme) checklist for Systematic Reviews (CASP Checklist (casp‐uk.net)).

A QuADS score of ≤13 was considered low, 14–29 medium and 30+ high quality. For CASP, the number of criteria not adequately met or unclear was used: 0–3 criteria = high, 4–6 = medium and 7–10 = poor quality. The AACODS uses ‘YES’, ‘NO’ and ‘Unclear’ criteria; papers successfully addressing all six categories were deemed high, those where 1–2 categories were not met or unclear as medium and papers where this was the case in more than two categories as low quality. No papers were excluded based on quality appraisal, rather this was considered during data synthesis and reporting.

### 3.7 Data abstraction

Data extracted included: year of publication, country, design, methodology and sample, participant characteristics (professional group), setting, outcomes of interest and reported definition of leadership (available from authors). A 10% sample of extracted data was independently reviewed for accuracy, completeness and consistency.

### 3.8 Data synthesis

A description of the study characteristics was developed, followed by thematic analysis (Braun & Clarke, 2021), where studies were organized to explore data patterns. All included papers were independently mapped against the Global Strategic Directions for Nursing and Midwifery 2021–2025 (WHO, 2021b). Studies were also scrutinized for leadership definitions, theoretical underpinning and barriers and facilitators regarding the nursing and midwifery contribution to leading DH policy and practice.

Ethical approval was not required. All decisions at each stage involved two independent reviewers, followed by discussion and review of any discrepancies by a third reviewer if needed to achieve consensus. All decisions were overseen by the wider review team and study steering group.

## 4 FINDINGS

### 4.1 Study characteristics

Table 2 summarizes the 24 papers included in the review. These comprised 6 empirical studies (Brommeyer & Liang, 2022; de Raeve et al., 2017; Hussey et al., 2021; Laukka et al., 2022; Peltonen et al., 2019; AMIA, 2022), 3 reviews (Bakker et al., 2023; Burgess & Honey, 2022; Ingebrigtsen et al., 2014), and 15 grey literature papers (Agnew, 2022; Ahonen et al., 2016; Ariosto et al., 2018; Bartz, 2014; Honey & Westbrooke, 2016; Hussey et al., 2015, 2017; Nurses Contribution to Swedish eHealth Strategy, 2012; ONMSD, 2022; Remus, 2016a, 2016b; Remus & Donelle, 2019; Sadoughi et al., 2016; Shi et al., 2016; Troncoso & Breads, 2021). Thirteen (54%) of the 24 were assessed as high, 7 (29%) as medium and 4 (18%) low quality (see File S1). Geographically, 12 papers concerned 9 individual nations (50%), 3 (14%) involved multiple countries (Europe, all‐Ireland, UK) and 7 were of global scope (30%).

**TABLE 2:** Summary of papers included in the review (*n* = 24).

Author	Title	Year	Country	Setting	Aims/objectives	Methodology	Participants (no. and professional group)	Quality assessment	Leadership definition	Global strategic directions for N&M domains addressed	Key findings
Agnew	Digital engagement in nursing: the benefits and barriers	2022	UK	NHS	Looks at the role of nurses in developing digital healthcare over the last 5 years	Opinion paper	Nurses	High	None.	Education Leadership Service Delivery	Barriers to engagement such as lack of resources, lack of nursing leadership in technology and digital innovation engagement
Ahonen et al.	The development process of e‐health strategy for nurses in Finland	2016	Finland	Healthcare in Finland	To describe nurses' contribution to the national strategy	Multiprofessional triangulation. Phase 1 group discussion Phase 2 integrative review Phase 3 joint virtual writing Phase 4 open web‐based questionnaire to FNA and other specialists	Expert panel *n* = 10 drawn from nursing practice/research/education/admin. Questionnaire *n* = 13	High	Refers to ‘nursing leaders’ and ‘strong’ leadership but no definition or theoretical underpinning. Describes what might be interpreted as leadership role of Finnish Nursing Assoc in implementing the strategy but not explicitly identified as such	Education Leadershi	Identified themes of the e‐health strategies: role of client nursing practice ethical aspects education and e‐health competencies nursing leadership knowledge management research and development
Ariosto et al.	Population health: a nursing action plan	2018	USA	Population Health in USA	To build capacity for population health.	Panel discussion.	AMIA Nursing Informatics Working Group panel comprising ‘opinion leaders’ who are nurses working in informatics.	High	None	Education Service Delivery	Provides recommendations for actions on population health in practice, education, policy and research
Bakker	Nurses' roles in mHealth app development: Scoping review	2023	Global	Nursing practice	To better understand nurses' role in mHealth app development	Scoping review	Nurses	High	None	Leadership Service Delivery	Nurses are involved in all stages of app development but mostly in requirement gathering and evaluator phases and least/rarely in designing apps and content or as research experts, patient advocates or informaticians. Nurses have a key role to play in addressing digital intervention failure due to misalignment between apps and existing workflows and clinical processes which leads to increased workload, and cumbersome work processes, which leads to burnout and nurses leaving the profession
Bartz	Leadership strategies for improved nursing synergy between informatics and telehealth	2014	Global	Nursing practice	To characterize the similarities and differences between informatics and telehealth from a nursing perspective. To advocate for leadership strategies that would bring the strengths of each group to a more synergistic, collaborative professional model for the benefit of both groups	Opinion paper comparing two separate yet often conflated areas of practice	Informatics and telehealth nurses	Medium	No clear definition but text mentions some elements of/implicitly implies transformational, authentic and collaborative leadership theory	Leadership	Informatics nurses and telehealth nurses have similar issues, which could benefit from collaboration and give them both a stronger voice. However, the two roles have their own areas of practice Proposes leadership is required to bridge the gap between telehealth and informatics Nursing
Brommeyer and. Liang	A systematic approach in developing management workforce readiness for digital health transformation in healthcare	2022	Australia	Australian digital health policy, Australian health service management postgraduate programme	To gain understanding and learn how to overcome the current hurdles in developing a health management workforce to maximize the benefits of digital health transformation	Three‐step, systematic approach: documentary analysis of Australian digital health policy Australian Health Service Management postgraduate programme analysis scoping review of international literature	N/A	Medium	Refers to role of health service leaders/managers and leading/managing—not defined or differentiated	Education Leadership	Urgent need to incorporate digital health‐related competencies in the existing training curriculum for health service managers. Importance of short‐term, targeted training in developing a health management workforce that is digital health ready, National collaboration is necessary to articulate more coordinated, consistent and coherent policy guidelines to foster digital health and workforce development
Burgess; M. Honey	Nurse leaders enabling nurses to adopt digital health: Results of an integrative literature review	2022	Global	Healthcare and e‐health	To synthesize the research exploring how nurse leaders can develop digital capability in the nursing workforce using the research question: ‘How do nursing leaders enable hospital nurses to adopt and use digital health technology?’	Integrative review	Nurses	Medium	Refers to transformational leadership and describes some elements of this	Education Leadership Service Delivery	Three main themes were identified: Connecting the digital and clinical worlds Facilitating digital practice development Empowering nurses in the digital health world
De Raeve et al.	Enhancing the provision of health and social care in Europe through e‐health	2017	Europe Health & Social Care	ENS4Care project	To establish evidence‐based guidelines to enable implementation of e‐health services in nursing and social care	Cross‐sectional, online, questionnaire survey of health professionals from 21 countries. Analysis used descriptive and summary statistics and content analysis of free‐text responses	Survey participants were described as professionals (*n* = 111, 91%), most of them were nurses, while nine practices (9%) were also submitted by service users and carers	Medium	Refers to ‘strong leadership’—not defined/described	Education Jobs Leadership Service Delivery	Five evidence‐based consensus statements for key steps and considerations for the deployment of e‐health services at different levels of enablement
Honey and Westbrooke	Evolving national strategy driving nursing informatics in New Zealand	2016	New Zealand	Healthcare and e‐health	Explores New Zealand health strategy highlights implications for nursing informatics and link to education and practice as: best use of technology and information, fostering and spreading innovation and quality improvements	Overview and review of NZ policy in e‐health	N/A	High	Refers to ‘strong leadership’ but has no definition and does not always specifically focus on/mention of nurses	Education Leadership Service Delivery	New Zealand and other countries need strong nursing leadership to sustain the nursing voice in policy and planning and ensure nurses develop the required informatics skills
Hussey et al.	Nursing Informatics and leadership, an essential competency for a global priority: e‐health	2015	Ireland	Nursing practice	Advocates for the growth of nursing informatics competencies as one method that can be used to support nursing leadership globally to address the challenges ahead	Opinion paper on the development of e‐health and the role nurses and nurse leaders have in influencing it	N/A	High	None.	Education Leadership Service delivery	Advocates the growth of nursing informatics competencies as one method that can be used to support nursing leadership globally to address the challenges ahead
Hussey et al.	ICNP (R) R&D Centre Ireland: defining requirements for an intersectoral digital landscape	2017	Ireland	Health and social care	To document Dublin City University's new research and development centre for International Classification for Nursing Practice (ICNP®) in Ireland	Process paper—ICNP research and development centre set up. Summary of the first year and how the initial activities link to development of global e‐health policy	N/A	High	No definition. Some of the text could be interpreted as leadership actions/leader priorities but not explicitly presented as such	Leadership Service delivery	Need to establish a platform to engage technical and human practices in advancing integrated care agendas—where both practice issues could be considered and solutions sought. Produced five action plans: (ICN, 2023) user group formation; (United Nations, 2016) research and development work plan; (UN, 2015) project management; (WHO, 2021a) education and training and (Krick et al., 2019) dissemination
Hussey et al.	A knowledge graph to understand nursing big data: case example for guidance	2021	Ireland	Centre of Integrated Care at Dublin City University, Ireland	Focuses on health informatics standards and translation of nursing knowledge to advance nursing theory through a nursing knowledge graph (NKG)	Co‐participatory study using design science methods—‘Open Innovation 2.0’	N/A	Low	Refers to nursing leaders but no definition/description	Education Service Delivery	Uses knowledge graph research development underpinned with Open Innovation 2.0 methodologies and design science research to enable review of data and connect diverse knowledge and new insights on service delivery
Ingebrigtsen et al.	The impact of clinical leadership on health information technology adoption: Systematic review	2014	Global	Healthcare and e‐health	To examine evidence of associations between clinical leadership and successful information technology (IT) adoption in healthcare organizations.	Systematic review	Nurses	High	Describe/discuss attributes of leaders and some role examples—no definition or leadership theory explicit	Education Leadership Service Delivery	Makes connections between the attributes of clinical leaders and IT adoption
Laukka et al.	Leadership in the context of digital health services: A concept analysis	2022	Finland	e‐health	To define and clarify the concept of leadership in the context of digital health services using Walker's and Avant's concept analysis model	Concept analysis	N/A	Medium	Proposes definition of and framework for e‐leadership. 2 of 23 papers in their review defined leadership	Education Leadership Service Delivery	Nursing leadership in the context of e‐health is ill‐defined and leaders who are competent and engaged in digital health appeared to enhance services
ONMSD	Digital nursing and midwifery (ONMSD)	2022	All Ireland	Health and Social Care	To create a 10‐year plan to transform health and social care services. Putting in place a modern digital health infrastructure is a key enabling strategic action to realize this transformation	All Ireland policy document	Nurses and midwives	High	No formal definition of leadership	Education Leadership Jobs Service Delivery	The National Nursing and Midwifery Digital Health Capability Framework–created to: define the digital health knowledge, skills and attitudes required for professional practice complement existing individual knowledge, skill and attitudinal frameworks provide a solid basis for tailored learning
Peltonen et al.	Emerging professionals' observations of opportunities and challenges in nursing informatics	2019	Global	Healthcare	To inform how to advance nursing informatics at local, regional and global levels	Content analysis of panel discussion	International panel organized by IMIA‐NI‐SEP group	Low	No definition.	Education Leadership Jobs Service Delivery	Recommended strategies for nurse leaders to improve: collaboration to build stronger infrastructure for NI education, research and practice improved visibility and appreciation of NI dissemination of evidence of NI in various health settings
Remus	The Big Data Revolution: Opportunities for Chief Nurse Executives	2016a	Global	Healthcare and e‐health	Focus on CNE informatics competency and nursing knowledge development as it pertains to the big data revolution. With the paper's aim of showing how CNEs armed with informatics competency can harness the full potential of big data offering new opportunities for nursing knowledge development in their clinical transformation roles as e‐health project sponsors	Opinion paper	Nurses, particularly focus on Chief Nursing Executives	High	Makes explicit links between e‐leadership and transformational theory of leadership	Education Leadership Service Delivery	Chief Nursing executives are ideally placed to be transformative leaders in digital healthcare, while also offering new opportunities for nursing knowledge development
Remus	Advancing the Digital Health Discourse for Nurse Leaders	2016b	Global	Healthcare and digital healthcare	To look at challenges to nursing leadership around their limited competency in informatics and their inability to influence digital health	Opinion paper	Nurse leaders	High	No definition of leadership in digital health—through maps transformational leadership theory onto characteristics needed to be a leader in the digital age	Education Leadership Service Delivery	Applies transformational leadership theory to explain how informatics competencies enable CNEs to become transformational nursing leaders in digital health allowing them to lead integrated, high‐quality care delivery using EBP. Informatic competent nurse leaders could also be in a position to drive organizational investment in technology and innovation
Remus and Donelle	Big data: why should canadian nurse leaders care?	2019	Canada	Canadian healthcare and e‐health	As in Remus (2016a, 2016b) : To discuss the need for CNEs to have informatics competency and the potential that this would have	Opinion paper	Nurses. Focus on Chief Nursing Executives	High	None. However strong message that nursing leaders need informatics competency to be effective in practice	Education Leadership Service Delivery	Canadian CNEs with informatics competency could impact Canadian healthcare by influencing clinical information systems ([CISs]) to support evidence‐based practice decisions and guide new health policy. Nurses input vast amounts of data to get minimal return but “uncovering” new evidence could provide new opportunities for nurse leaders by offering robust, electronic tools, which support informed decision‐making
Sadoughi and Azadi	Nurses' contribution to health information technology of Iran's 2025 health map: a review of the document	2016	Iran	e‐Health	To investigate nurses' contribution to health information technology of Iran's 2025 health map	Policy review	N/A	Low	Refers to improving leadership of nurses in e‐health services—but no definition of leadership	Leadership Education Service delivery jobs	Nurses' contribution is not clearly stated in the strategy. Recommends more attention is paid to contribution of nurses in further actions to revise Iran's e‐health strategy. Asserts a need for benchmarking nurses' role against the successful experiences of other countries in revising and developing Iran's e‐health strategy
Shi et al.	Leading the development of our new nursing information system with the TIGER‐based Taiwan Model	2016	China	Developing a nursing information system	To document how it improved nursing information system by (ICN, 2023) promoting the informatics competency, (United Nations, 2016) setting up and educating the core task force, (UN, 2015) training professional informatics nurses, (WHO, 2021a) redesigning the usability of current NIS and (Krick et al., 2019) building the leadership team composed of stakeholders and consultants from Taiwan	Process paper	Taskforce of 1159 nurses took part in consultation process. and later competency training in informatics	Low	Refers to nurses leading but no definition/description.	Education Leadership Service Delivery	Learning came from consultation with colleagues from Taiwan in which clinical nurses build up their informatics competency and lead the design and development of NIS
Strudwick	Opportunities and challenges to enhance the value and update of Chief Nursing Informatics Officer (CNIO) roles in Canada: A qualitative study	2022	Canada	Healthcare	To explore the experiences, perceptions and needs of current health system leaders in CNIO and CMIO roles to influence future uptake and perceived value of these roles	Interview study	Chief Nursing Informatics and Chief Medical Informatics Officers (*n* = 10)	Medium	No definition of leadership	Education Leadership Jobs Service Delivery	Importance of partnership working between CNIO and CMIO. Key roles in bridging the clinical‐technical divide to address the implementation to benefits realization gap associated with digital interventions. Need coordinated education and career strategy for these roles. Need to increase awareness of these roles and their value. Need to embed informatics competencies for nurse leaders in core leadership curricula
Törnvall	Nurses’ contribution to Swedish e‐health strategy	2012	Sweden	Healthcare	To describe nurses' contribution to the establishment of national cooperation concerning e‐Health development in health and social care.	Policy review on nurses contribution to e‐health development	Nurses	High	No definition of leadership though describes leadership role of Swedish Society of Nursing in engaging in IT strategy/policy	Education Leadership Service Delivery	Swedish Society of Nursing priorities in influencing e‐health strategy: Patient voice clearly articulated Nationally compatible patient health record Intervention to facilitate patients' and caregivers' accessibility to information and e‐prescriptions Swedish Society of Nurses appears to drive forward nurse empowerment and involvement in technology
Troncoso and Breads	Best of both worlds: digital health and nursing together for healthier communities	2021	USA	Digital healthcare	To give a set of practical recommendations to the nursing and digital health communities to achieve a common vision of nurses fully engaged with and leading digital health solutions for universal health coverage	Opinion paper	Nurses	Medium	No explicit definition. Refers to systemic changes requiring engagement, leadership and championing from nurses	Education Leadership Service Delivery	Nursing and digital health communities have much to gain from each other and can be stronger together. Offers practical recommendations for both communities to optimize mutual efforts towards achieving UHC: Increase nurses' access to digital technologies and digital skills Promote nursing collaboration in design, monitoring and evaluation of digital health Develop principles for digital technology use within clinical settings Invest at the intersection of nursing and digital health for high rates of return and development of new models of care Promote the vision of a digitally enabled nurse

We used the terms ‘nurses’ and ‘midwives’ throughout the review process to ensure both professional groups were included. One paper (ONMSD, 2022) consistently used both terms throughout, and one other (Hussey et al., 2017) referred to both groups, although inconsistently. We therefore use nurses/nursing throughout this paper to include nurses/nursing and midwives/midwifery.

**FIGURE 2:**
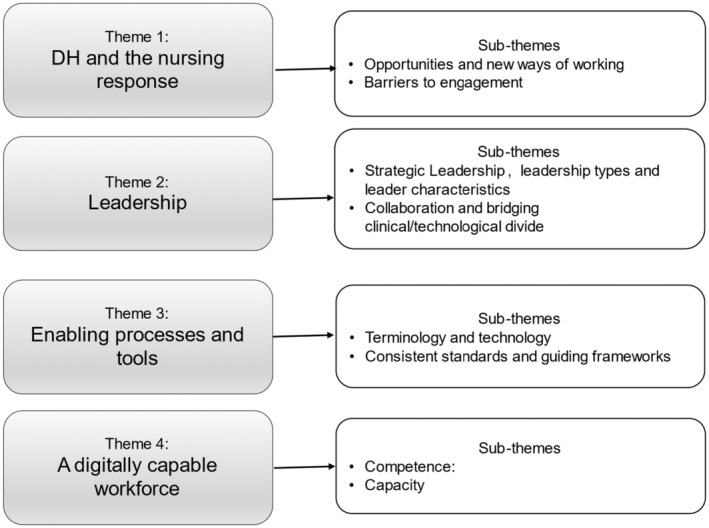
Theme map.

Four themes were identified. The first two: ‘Digital health and the nursing response’ and ‘Leadership’ addressed the role of nursing in DH. Themes 3 and 4: ‘Enabling processes and tools’ and ‘Developing a capable workforce’ addressed the infrastructure required for nursing leadership in DH. Figure 2 outlines these themes and associated sub‐themes, and the theme density map (File S2) shows the papers comprising each theme/sub‐theme.

### 4.2 Theme 1: DH and the nursing response

Twenty of the 24 papers addressed this topic, which comprised two sub‐themes: ‘Opportunities and new ways of working’ and ‘Barriers to engagement’.

#### 4.2.1 Opportunities and new ways of working

Twenty‐one papers comprised this sub‐theme, 12 of these were high, 7 medium and 2 of low‐quality. The findings indicate nurses can play a pivotal role in transforming healthcare (Remus, 2016a), by influencing systems and solutions to support person‐centred, coordinated care now and for the future (ONMSD, 2022), but they need the right knowledge and skills (de Raeve et al., 2017). For example, as telehealth becomes mainstream, nurses will increasingly use remote consultations to ensure a timely, cost‐effective service (Burgess & Honey, 2022; Honey & Westbrooke, 2016; Hussey et al., 2015; Troncoso & Breads, 2021). However, while there may be benefits to remote working, it cannot fully replace face‐to‐face nursing care (de Raeve et al., 2017) and can inadvertently create new risks to patient safety, for example, through virtual wards (Agnew, 2022).

Twenty‐one of the 22 papers highlighted the vital contribution nurses make, including through their unique knowledge, skills and access to information, and how this can be harnessed to drive successful digital innovation (Ahonen et al., 2016; AMIA, 2022; Bakker et al., 2023; Troncoso & Breads, 2021). The principles underpinning the concept of the digital patient are highly consistent with established nursing theories and models (Bartz, 2014; Hussey et al., 2015). This affords nurses a pivotal role in improving care quality while guiding people through technological advancement and health system changes (Troncoso & Breads, 2021). The systemic changes involved in the digital transformation of healthcare require nurses' engagement, leadership and championing (Troncoso & Breads, 2021). These, in turn, rely on having technology and systems that align with nurses' professional values and are fit for purpose within a digital practice setting (Agnew, 2022; de Raeve et al., 2017; Hussey et al., 2017; Troncoso & Breads, 2021); for example, systems providing real‐time, clinical information in a usable format (Remus, 2016a), which increases patient safety (Agnew, 2022).

Several papers discussed the changing nature of healthcare and the opportunities this presents for nursing, with the most recent (Agnew, 2022; Brommeyer & Liang, 2022; Burgess & Honey, 2022; Hussey et al., 2021; Laukka et al., 2022; ONMSD, 2022; Troncoso & Breads, 2021) acknowledging the impact of COVID‐19 in expediting the growth of DH and associated changes in care delivery. Generally, high levels of household internet access have led to increased use of patient portals (Honey & Westbrooke, 2016) and telehealth consultations becoming a convenient and timely care alternative (ONMSD, 2022). Workforce challenges have led to demands for new ways of working (de Raeve et al., 2017), through a ‘big data revolution’ (Remus & Donelle, 2019), and developments in artificial intelligence and predictive analytics (Agnew, 2022), driven by EU and WHO policy to enable new models of care (Ahonen et al., 2016). Furthermore, national nursing bodies such as the UK Royal College of Nursing recognize the future of nursing involves a digital component, requiring support for the whole profession to practice in new ways (Agnew, 2022). Internationally, the WHO Framework for Integrated Care provides supportive scaffolding for DH transformation, but the pace of progress differs across the globe (Hussey et al., 2017).

Technology has changed the way many nurses work across all practice settings (ONMSD, 2022), using an array of electronic assessment, diagnostic and acuity tools. This highlights the knowledge and experience of nurses in using technology despite often inadequate usability and interoperability, resulting from limited opportunities to influence the design and decision‐making processes that drive DH (Troncoso & Breads, 2021). Nurses should be considered key partners in designing, planning, delivering and evaluating DH services that do more than just address workforce gaps (Hussey et al., 2021). Nurse leaders then must recognize the potentially powerful position this presents for the profession in delivering future models of care and advancing the DH agenda (Hussey et al., 2015; Remus, 2016b). Focusing specifically on big data illustrates how data science can evolve nursing knowledge to generate practice‐based evidence (Remus & Donelle, 2019). This provides opportunities for nurses to use ‘real‐time’ evidence in practice, addressing the WHO call for practitioners to use digital tools to improve health outcomes and deliver on the UN SDGs (Troncoso & Breads, 2021), while working in partnership with patients to support appropriate, informed decision‐making and health literacy (ONMSD, 2022).

Nurses have an important role in sustainable healthcare development, which requires clarification of their contribution to national and international strategy development alongside other key stakeholders (Sadoughi et al., 2016). Three papers highlighted further challenges for nurses in maximizing the benefits of digital technology. These included how to empower and work in partnership with healthcare consumers to enable realistic goal setting, using digital methods such as biometrics to support healthy living monitoring (de Raeve et al., 2017; Honey & Westbrooke, 2016), enabling citizens to add to their health records and compare care providers (Nurses Contribution to Swedish eHealth Strategy, 2012). Globally, however, nurses and citizens have variable access to the internet and digital technologies (ONMSD, 2022) underlining the need for awareness of DH challenges as well as opportunities (de Raeve et al., 2017).

Several papers argued that nurses are already engaged in digital transformation. Furthermore, nursing engagement in big data science has been well‐referenced in the nursing literature over the last two decades (Hussey et al., 2021). One paper (Agnew, 2022), citing the UK RCN ‘Clever Together’ report, argued that commonly held perceptions of nurses' reluctance or inability to use technology are unfair because they have not been equipped with the basic tools; nor has their contribution been appropriately recognized (Sadoughi et al., 2016).

Addressing such challenges requires cross‐functional teamwork involving clinical and technical expertise (AMIA, 2022; Hussey et al., 2017). This is particularly important as nursing experiences a paradigm shift from technology designed to collect data at individual points of care within organizations, to data collected and coordinated across a person's health trajectory and the associated potential safety risks of this, including errors in data collection and workflow management processes (Ariosto et al., 2018).

#### 4.2.2 Barriers to engagement

Twenty‐one papers reported barriers to nurses' engagement in digital transformation; 13 of which were high quality, 7 medium and 1 of low quality. Barriers fell into four categories: perceived risks, negative impact on workflow/workload, limited opportunities for involvement at policy and development levels and unsupportive organizational cultures. Perceived risks to the trust‐based, therapeutic relationship and patient perceptions were reported (Agnew, 2022) and linked to concerns over privacy and data protection (Bartz, 2014; Troncoso & Breads, 2021).

Five papers identified the impact of digital technology on workflow as a barrier. Issues included an already heavy workload (Agnew, 2022; Bakker et al., 2023; Burgess & Honey, 2022), particularly as the introduction of new technology rarely resulted in the discontinuation of previous practices (Burgess & Honey, 2022). Digital innovation also added new responsibilities and tasks such as routine monitoring of the impact on health outcomes and workflow (Burgess & Honey, 2022; Troncoso & Breads, 2021), therefore nurses need resources such as time and infrastructure (Ahonen et al., 2016), adequate equipment, systems and support for digital skills development (Agnew, 2022; Ariosto et al., 2018; Brommeyer & Liang, 2022; Burgess & Honey, 2022; de Raeve et al., 2017; Hussey et al., 2015, 2021; ONMSD, 2022; Remus & Donelle, 2019).

The third barrier concerns limited opportunities for nurses' involvement in DH policy and strategy development due to inadequate enabling mechanisms (Troncoso & Breads, 2021) and the little return they often see for the time and effort invested when they do (Agnew, 2022; Remus & Donelle, 2019). This creates a vicious circle, meaning the nursing voice is often not evident in DH design, implementation and evaluation (Burgess & Honey, 2022; Troncoso & Breads, 2021), despite co‐design being a priority (Honey & Westbrooke, 2016). The exception is the development and contribution of NI as a discreet nursing discipline, although the siloed and high‐income country‐based nature of this has attracted criticism (Troncoso & Breads, 2021).

Culture was the fourth main barrier to nurse engagement in digital transformation, along with the role nurse leaders play in addressing this (Burgess & Honey, 2022; Ingebrigtsen et al., 2014; Laukka et al., 2022). One paper called for nursing to redefine its professional culture regarding DH and increase collaboration among nurses, system developers and patients (Agnew, 2022); while another highlighted the leadership characteristics and behaviours needed to facilitate culture change (Burgess & Honey, 2022). Strategies proposed included developing small technology user groups and connecting these to larger national/international groups (Hussey et al., 2017).

### 4.3 Theme 2: Leadership

This comprised sub‐themes: *‘*Strategic leadership: Leadership types and leader characteristics’ and ‘Collaboration and bridging the clinical/technological divide’.

#### 4.3.1 Strategic leadership: Leadership types and leader characteristics

Seventeen papers comprised this sub‐theme; 10 of these were high, 6 medium and 1 of low quality. One paper provided a leadership definition, confirming that: ‘Few authors provide an official definition for leadership, or its closely related terms, in the context of digital health services…’ (Laukka et al., 2022, p. 2766). While a further 8 (Ahonen et al., 2016; Bartz, 2014; Burgess & Honey, 2022; Hussey et al., 2017; Ingebrigtsen et al., 2014; ONMSD, 2022; Remus, 2016a, 2016b) described characteristics of nurse leaders; the remaining 14 papers provided neither. Similarly, papers did not define the term ‘nurse leader’; some referred specifically to nurse managers, although leadership can be exercised by all nurses as a core function of clinical practice (Burgess & Honey, 2022).

Eight of the 22 papers (Brommeyer & Liang, 2022; Burgess & Honey, 2022; Hussey et al., 2015; Ingebrigtsen et al., 2014; ONMSD, 2022; Remus, 2016a, 2016b; Remus & Donelle, 2019) argued that today's nurse leaders need to have digital capability to underpin their leadership credibility; and effectiveness in influencing change and the development of appropriate health systems and tools (Brommeyer & Liang, 2022). Digital capable leaders can assess the organizational need for digital expertise and develop new DH roles (Peltonen et al., 2019; Remus, 2016b); contribute to large‐scale design science initiatives for sustainable healthcare (Hussey et al., 2021); and lead corporate technological discussions, advocating for the design and integration of digital solutions that meet nursing needs (AMIA, 2022; Remus, 2016a, 2016b).

Four papers (Bartz, 2014; Laukka et al., 2022; Remus, 2016b; Troncoso & Breads, 2021) specifically highlighted the need for a new kind of leadership within a digital environment, one which avoided traditional autocratic command and control approaches (Remus, 2016a, 2016b) that contributed to previous technology implementation failures (Ingebrigtsen et al., 2014). This leadership advocates for useful DH solutions for nurses (Remus & Donelle, 2019) and is context sensitive (Brommeyer & Liang, 2022), creating a positive work culture (Burgess & Honey, 2022), a team science approach (Remus & Donelle, 2019), encompasses multifaceted capabilities, builds trust and supports ethical practice, while maintaining suitable boundaries such as those related to resource allocation and decision‐making responsibilities (Bartz, 2014). The limited research on current approaches to DH leadership has led to calls for the appropriateness of transformational leadership, especially when combined with digital capability to facilitate nurses' pivotal transformation role, to be confirmed (Burgess & Honey, 2022). This is in addition to the recommended reconceptualization of DH leadership and use of the term e‐leadership, first introduced in the 1990s (Remus, 2016a), in nursing research and practice (Laukka et al., 2022).

The importance of nurses in key leadership roles being fully engaged with DH services (Laukka et al., 2022) and informatics competent (Remus, 2016a; Remus & Donelle, 2019) was emphasized as necessary for them to effectively discharge their strategic advocacy role and develop future DH leaders and capability (Honey & Westbrooke, 2016). This includes advocating for resources and investment to support sustainable transformation, including education and training (Ahonen et al., 2016; Burgess & Honey, 2022; Ingebrigtsen et al., 2014; Remus, 2016a); building time and learning opportunities into everyday work to generate a supportive learning culture (Burgess & Honey, 2022); enabling the introduction of specialist roles (Remus, 2016a); and providing expert advice to professional regulatory bodies regarding DH practice standards (Remus, 2016b).

Although most of the literature focused on nursing leadership enabling the implementation and adoption of digital innovation, nurse leaders should be involved in wider national discussions in addition to enabling the development, implementation and use of digital innovations at an organizational level (Peltonen et al., 2019). This requires that nurses are advocating for and facilitating the elements of evidence‐based nursing care (Remus & Donelle, 2019).

actively involved in… leadership, policy development and advocacy for digital health at individual, local and national levels (ONMSD, 2022, p. 9),

As complex interventions, digital innovations require a strategic, multifaceted approach involving inter‐ and intra‐organizational collaboration between health, education/professional bodies and governments (Brommeyer & Liang, 2022), alongside organizational change on multiple levels (Laukka et al., 2022). The positive strategic impact of nursing leaders engaging staff, being visible, providing coaching and ensuring frontline nurses are heard by relaying their feedback to technology managers was also highlighted (Bartz, 2014). Specific strategies for achieving this, such as ‘super users’ or digital coaches, were suggested (Burgess & Honey, 2022) to enhance nurse leaders' capacity.

#### 4.3.2 Collaboration and bridging the clinical/technological divide

Seventeen papers comprised this sub‐theme; nine of these were high, six medium and two of low quality. The emergence of Chief Nursing Informatics Officers (CNIOs) has been supported by Chief Medical Informatics Officers who recognize the need for these groups to partner in redesigning clinical workflows to enhance care delivery in the USA (Remus, 2016a) and Canada (Bakker et al., 2023). However, the informatics–telehealth gap within nursing and the need for these two disciplines to develop a collaborative professional model that benefits both were also reported (Bartz, 2014).

Furthermore, the role of nurse leaders in bridging the digital and clinical divide was identified as a current global leadership challenge (AMIA, 2022; Bakker et al., 2023) alongside a lack of guidance for such collaboration, which is hindering both sectors' ability to influence the future of DH for universal health coverage (Troncoso & Breads, 2021). Where nurses do contribute to strategy development, their role is not always well‐defined or visible (Sadoughi et al., 2016). However, nurse leaders who create clinical–digital links facilitate digital transformation in practice; by acting as mediators between implementation priorities and clinical workflow realities, intervening to reduce unintended consequences that may compromise the use of digital solutions (Burgess & Honey, 2022; Ingebrigtsen et al., 2014; Remus, 2016b).

Lack of support from nursing leaders was reported as contributing to why DH services implementation often fails (Burgess & Honey, 2022; Laukka et al., 2022; Remus, 2016a). More specifically, a lack of digital nursing leadership was a major barrier to nurse engagement (Agnew, 2022; Peltonen et al., 2019). Reported nurse leader deficits concerned: NI and data science (Peltonen et al., 2019; Remus, 2016a; Remus & Donelle, 2019), awareness of their leadership role in digital transformation (Brommeyer & Liang, 2022), awareness of general nursing terminology and importance of its use (Remus & Donelle, 2019), and the benefits of technology (Brommeyer & Liang, 2022). Exemplars of nursing leadership in DH policy and strategy development were highlighted, including the development of Australia's DH policy (Brommeyer & Liang, 2022), Sweden's NI strategy (Hussey et al., 2015), China's nursing information system (Shi et al., 2016) and the national e‐health programme for Ireland (Hussey et al., 2017). Internationally, the role of nursing leadership groups such as the European Federation of National Nursing Associations in developing clinical guidelines based on best practice evidence regarding DH services from across EU member states was highlighted (Hussey et al., 2015).

### 4.4 Theme 3: Enabling processes and tools

This comprised sub‐themes: *‘*Terminology and technology’ and ‘Consistent standards and guiding frameworks’.

#### 4.4.1 Terminology and technology

Twenty‐one papers comprised this sub‐theme; 11 of these were of high, 7 medium and 3 of low quality. DH transformation is fast developing, yet the associated terminology, concepts and roles remain poorly understood (Peltonen et al., 2019). Multiple terms are used to describe ‘digital health’ and ‘digital nursing’ with 9 of the 22 papers providing definitions of these terms. These included: DH (Burgess & Honey, 2022; Troncoso & Breads, 2021) and e‐health (Ahonen et al., 2016; Bartz, 2014), with two (Ahonen et al., 2016; Burgess & Honey, 2022) drawing on relevant WHO definitions. Other definitions included: digitization (Laukka et al., 2022), big data (Remus, 2016b; Remus & Donelle, 2019), data science (Remus & Donelle, 2019), informatics and NI (Bartz, 2014), informatics literacy (Remus, 2016b; Remus & Donelle, 2019) and NI literacy and telehealth/telenursing (Bartz, 2014). Referring to DH practice, one paper (ONMSD, 2022) defined digital professionalism and another e‐leadership (Remus, 2016b). Finally, the concept of digital wisdom was proposed (Bartz, 2014) as a better illustration of what was required.

Successful digital innovation relies on systems being fit for purpose, accessible and with good connectivity (AMIA, 2022; de Raeve et al., 2017), yet limited access to digital technology and skills are the biggest problems facing nursing globally (Troncoso & Breads, 2021) and a major reason for limited uptake of digital solutions (Brommeyer & Liang, 2022). Commonly reported issues included: too few computers; password difficulties; lack of support; duplicating records (paper and online); overcomplicated software and dated hardware; and poor connectivity (Agnew, 2022). The impact is duplication, increased documentation and alert fatigue (Remus, 2016a); inefficient systems resulted in wasted time (Agnew, 2022) and reduced effectiveness and efficiency (Brommeyer & Liang, 2022; Shi et al., 2016). This leaves nurses disempowered (Burgess & Honey, 2022) and being used as ‘data entry clerks’ (Remus, 2016a); fuelling perceptions that technology is more about organizational management and control than enabling clinical work (Agnew, 2022), which can drive disengagement (Burgess & Honey, 2022). Additionally, introducing new technologies that do not reflect practice realities, or meet nurses' clinical or workflow needs, creates additional work and disrupts workflow efficiency (Ariosto et al., 2018; Bakker et al., 2023). Furthermore, nurses spend considerable time inputting data, which does not often reflect their care contribution or enable them to improve care quality (Agnew, 2022). Therefore, many papers (Agnew, 2022; Ahonen et al., 2016; AMIA, 2022; Bakker et al., 2023; Burgess & Honey, 2022; Hussey et al., 2015, 2017, 2021; Troncoso & Breads, 2021) argued that enabling greater nursing involvement in the design, implementation and evaluation of digital systems would improve engagement.

#### 4.4.2 Consistent standards and guiding frameworks

Nineteen papers comprised this sub‐theme; 11 of these were appraised as high, 5 as medium and 3 as low quality. They focused on the need for consistent DH policy, standards and education frameworks. These encompassed DH practice (job roles, licensure and technology); education (competencies and education standards); and governance to enable collaborative working and learning while maintaining security and safety concerning issues such as data anonymization and enforcement. The EU also recognized the potential risk of cyber violence/harassment associated with digital settings (Troncoso & Breads, 2021).

National infrastructures need to develop from disparate, siloed systems to integrated information‐sharing platforms (Ariosto et al., 2018; de Raeve et al., 2017; Honey & Westbrooke, 2016; Hussey et al., 2015, 2017, 2021; ONMSD, 2022; Remus, 2016a; Remus & Donelle, 2019). At the WHO regional level, Open Innovation 2.0 offers a new paradigm to advance innovation and shape Europe's digital future, with government, industry, academia and civil participants co‐creating structural changes (Hussey et al., 2021). Policy initiatives set out in the European Commission's Digital Agenda, which aim to ensure close cooperation between EU member states and different stakeholders, can act as the driving force for the implementation of e‐health (de Raeve et al., 2017). However, such national/regional infrastructure needs to be underpinned by international guidelines, such as the WHO guideline on digital interventions for health system strengthening (Brommeyer & Liang, 2022). This enables nations to learn lessons and best practices from each other (Sadoughi et al., 2016). A lack of global leadership, particularly in connecting the DH and nursing sectors, and guidance for collaboration hinders both (AMIA, 2022; Troncoso & Breads, 2021). Specifically referring to NIs, emerging international guidelines on roles, education and certification used to support standardized competencies were highlighted (AMIA, 2022; Bakker et al., 2023; Peltonen et al., 2019). However, developing international guidelines presents unique challenges as they need to apply to different countries that have differing degrees of autonomous practice and education systems.

Despite this, examples of national and international nursing bodies having developed frameworks to support digital transformation were identified (de Raeve et al., 2017; Remus & Donelle, 2019). A key aspect of service delivery relates to defining care requirements, specifically to support sustainable intersectoral healthcare. This requires considering how nursing‐sensitive language/terminology is best mapped to articulate the care requirements and processes to achieve optimal patient outcomes (Hussey et al., 2017) and capture the actual work of nurses (Agnew, 2022).

We identified several examples of practice standards supporting a consistent approach to DH development at the national/international level (AMIA, 2022; Bartz, 2014; Brommeyer & Liang, 2022; ONMSD, 2022). Furthermore, the ICN has a long‐established DH programme, supported by policies and strategies to advance nurses' knowledge and involvement in DH globally (Ahonen et al., 2016). This includes the ICN International Professional Standards for Telehealth nursing programmes (Bartz, 2014) and supporting the use of International Classification for Nursing Practice (ICNP; Ahonen et al., 2016), although nursing leaders need to focus on developing health informatics standards for structural interoperability (Bakker et al., 2023; Hussey et al., 2021). This is mirrored by a call for a stronger global NIs community to collaboratively build better infrastructure to guide education, research and practice (Peltonen et al., 2019). In addition to country‐based associations, organizations such as the ICN and the International Society for Telemedicine and eHealth (ISfTeH) provide designated networks offering structured support to nurses engaged in informatics and telehealth (Bartz, 2014). In summary, we found that nurses need to be able to access training, which is standardized nationally and internationally. However, current education provision is fragmented and lacks standardization, creating challenges around professional boundaries, licensure and job specifications.

### 4.5 Theme 4: A digitally capable workforce

Twenty‐one papers highlighted the importance of ensuring a digitally capable workforce. The findings are presented in two sub‐themes as necessary for achieving this: *Competence and Capacity*.

#### 4.5.1 Competence

Twenty‐one papers comprised this theme and highlighted the implications of digital transformation for nursing skills development. Thirteen of these were appraised as high, seven as medium and one as low quality.

Four papers (Ahonen et al., 2016; Honey & Westbrooke, 2016; ONMSD, 2022; Troncoso & Breads, 2021) noted that because of the growing use of DH technologies in nurses' daily roles, previously considered optional or specialist skills are now crucial for all practitioners. For example, every nurse has a role in informatics (Hussey et al., 2015) but success depends on staff understanding the significance of the socio‐technical dimensions of DH improvement (Hussey et al., 2017; ONMSD, 2022). Upskilling the workforce in this regard is a long‐term process requiring investment (de Raeve et al., 2017) and a holistic approach to developing the necessary workforce capabilities and system‐wide capacity needed (Brommeyer & Liang, 2022; Hussey et al., 2017, 2021); for example, by using nursing informaticians to act as catalysts and supporting capacity development in all nurses (Remus, 2016a).

Many papers addressed the education of nurses for digital practice. Nurses need support in assessing DH literacy in the communities they work with (de Raeve et al., 2017) and better support to access education to improve their digital knowledge and skills (Burgess & Honey, 2022), if new service delivery models are to be effective (Troncoso & Breads, 2021). Furthermore, nurses require basic digital skills and internet access to function effectively in day‐to‐day practice (Ariosto et al., 2018), therefore, this should be a core part of all health professional and clinical education (Burgess & Honey, 2022); because informatics competency is a core commodity for enabling nurses to support the digital patient (Hussey et al., 2015; Remus & Donelle, 2019).

Overall, the papers reviewed identified limited knowledge and a lack of guiding frameworks to support DH education, particularly concerning digital leadership (AMIA, 2022). An e‐leadership framework has been developed for further testing (Laukka et al., 2022), although there is no identified systematic, guiding or competency framework to support the education of healthcare managers on how to lead and manage the workforce through digital transformation (Brommeyer & Liang, 2022). Furthermore, digital competencies are not well‐defined or formally integrated into nursing education and practice (Troncoso & Breads, 2021).

Nurse leaders' role in facilitating and actively supporting staff to access education is crucial (Burgess & Honey, 2022; Remus, 2016b). This involves practicalities such as providing protected time for learning and access to digital coaching, as well as developing a learning culture. Furthermore, nurse leaders must recognize how their own beliefs, skills and attitudes towards digital innovation influence their teams (Ingebrigtsen et al., 2014). There is currently no comprehensive, measurable means of identifying e‐leadership in practice, although this may be partly achieved by using the Nursing Informatics Competency Assessment for Nurse Leaders (Laukka et al., 2022).

The literature calls for a tapestry approach to developing digital skills in the workforce. This should span under/postgraduate education and continuing professional development with depth based on different DH roles to maximize relevance and engagement (Agnew, 2022; Bartz, 2014; Nurses Contribution to Swedish eHealth Strategy, 2012; Peltonen et al., 2019). For example, the Australian Digital Health Agency roadmap identified eight staff profiles and associated digital capabilities across health workforce roles and contexts to enable this (Brommeyer & Liang, 2022). Several papers described current education provisions and recommendations to address gaps. These included providing a mix of mandatory/formal and optional learning, plus different formats, including on‐demand e‐learning/webinars, to increase access (Burgess & Honey, 2022). There is also an urgent need for short‐term/targeted programmes to maximize relevance and meet the needs of specific groups, such as those who already have post‐graduate qualifications; coupled with a systematic and universal framework for education and research; and generation of up‐to‐date evidence to guide the development of managers in the digital era (Brommeyer & Liang, 2022).

#### 4.5.2 Capacity

Capacity encompasses various elements, including time, equipment, job roles and cultural factors. Seventeen of the 22 papers identified workforce capacity as a critical factor for achieving the benefits of DH; 9 of these were high, 7 as medium and 1 as low quality.

Digital transformation involves complex, socio‐technical change at personal, organizational and system levels (de Raeve et al., 2017; Ingebrigtsen et al., 2014; Laukka et al., 2022). Interactions among technology, work processes and people mean that organizational readiness for change is crucial (Ingebrigtsen et al., 2014; Peltonen et al., 2019). It may involve disruption of usual practice, introducing new organizational structures (de Raeve et al., 2017), and redistribution of power and work between groups (Ingebrigtsen et al., 2014). DH development, therefore, must be adequately planned, resourced and prioritized alongside other investments (Burgess & Honey, 2022; de Raeve et al., 2017; Nurses Contribution to Swedish eHealth Strategy, 2012), based on an a priori cost analysis that includes not only factors associated with care production but also the risk to patient safety (Nurses Contribution to Swedish eHealth Strategy, 2012), potential negative impact on nurse/patient interaction (Troncoso & Breads, 2021), and necessary investment in nursing skills development and infrastructure (ONMSD, 2022).

The importance of an organizational infrastructure that supports and makes visible the nursing contribution to DH was a common thread. For example, the presence of a CNIO at the organization's ‘top table’ had a positive impact on IT adoption (Ingebrigtsen et al., 2014). Furthermore, partnership working between Chief Nursing and Medical Informatics Officers was highlighted to ensure workflow design is fit for purpose (AMIA, 2022; Remus, 2016a); manages population health complexities (Ariosto et al., 2018); and ensures the potential of technology‐based big data to guide real‐time, evidence‐based clinical decision‐making (Hussey et al., 2021; Remus & Donelle, 2019). Several studies have shown that leaders who established clear, formal structures for IT governance, via layers of multidisciplinary governance committees, were more likely to achieve successful outcomes (Ingebrigtsen et al., 2014).

Although new digital nursing roles (Remus, 2016a) and specialisms (Hussey et al., 2015) have been established in several countries, these vary globally, and the associated lack of recognition and development opportunities (AMIA, 2022) mirrors the development of other advanced nursing roles (Peltonen et al., 2019). This has led to calls for a career pathway for DH nurses (AMIA, 2022), with appropriate remuneration to reflect the additional responsibility (de Raeve et al., 2017). In addition, professional associations and regulatory bodies must note the need to clarify professional boundaries within which advanced professionals will employ DH solutions (de Raeve et al., 2017) and all nurses need knowledge of the full scope of their practice/licence for their role and context (Bartz, 2014). Furthermore, in addition to the need for supportive workforce policy and funding/resources at all levels, collaboration among universities, professional and healthcare organizations (Brommeyer & Liang, 2022), using strategies such as a common educational pathway, for example (AMIA, 2022; de Raeve et al., 2017), is also necessary to ensure workforce capacity.

Our findings highlight that NI education, certification and specialist roles, for example, are not well‐adopted globally (Peltonen et al., 2019). For Europe, the ENS4Care guidelines should enable leaders to identify and address the organizational changes needed and develop new workforce skills and roles to meet the challenge of DH (de Raeve et al., 2017). However, there is a need to translate competency statements into action items at policy and education levels to ensure nurses are ‘competent users and informed consumers’ (Peltonen et al., 2019). Currently, finding staff with the right qualifications for NI roles can be difficult (Peltonen et al., 2019), although supporting education and networking will help nurse leaders find relevant resources (Remus, 2016b). In terms of remaining gaps, the literature reviewed called for system‐wide capacity building within organizational systems and structures, aligned with the education and training offer (Brommeyer & Liang, 2022).

## 5 DISCUSSION

Digital technologies are changing the care landscape for patients and staff, and if well supported, can improve health outcomes and quality of care, while maintaining person centredness. This requires nursing leadership at all levels of policy and practice but a common thread throughout this review was recognition of the lack of a robust infrastructure to support strategic nursing leadership and calls for this to be addressed for maximum benefit to be realized from digital transformation on an interdisciplinary/inter‐sectoral and international scale.

Healthcare infrastructure can be conceptualized as comprising multiple elements, including the built environment, governing processes and systems and the people working within it. The Global Strategic Directions for Nursing and Midwifery (GSDSM) (WHO, 2021b) represents key global workforce policy designed to help countries achieve universal health coverage and population health goals by optimizing nursing practice through strategic actions to support infrastructure development. It sets priorities in four domains (education, service delivery, leadership and jobs), therefore, this discussion is structured using the GSDNM (WHO, 2021b) domains, and Table 3 provides an overview of the extent to which the eligible papers address these. This indicates that although a comparable number of papers address the *Education*, *Service Delivery and Leadership* domains, much less attention has been paid to the *Jobs* domain, with only 5 of the 24 papers addressing this.

**TABLE 3:** Papers mapped against the GSDNM (WHO, 2021a, 2021b, 2021c, 2021d) domains.

Author	GSDNM domains			
	Education	Jobs	Leadership	Service delivery
Agnew (2022)	x		x	x
Ahonen et al. (2016)	x		x	
Aristo et al. (2018)	x			x
Bakker et al. (2023)			x	x
Bartz (2014)			x	
Brommeyer and Liang (2022)	x		x	
Burgess and Honey (2022)	x		x	x
De Raeve et al. (2017)	x	x	x	x
Honey and Westbrooke (2016)	x		x	x
Hussey et al. (2015)	x		x	x
Hussey et al. (2017)			x	x
Hussey et al. (2021)	x			x
Ingebrigtsen et al. (2014)	x		x	x
Laukka et al. (2022)	x		x	x
ONMSD (2022)	x	x	x	x
Peltonen et al. (2019)	x	x	x	x
Remus (2016a)	x		x	x
Remus (2016b)	x		x	x
Remus and Donelle (2019)	x		x	x
Sadoughi et al. (2016)	x	x	x	x
Shi et al. (2016)	x		x	x
Strudwick et al. (2022)	x	x	x	x
Törnvall (2012)	x		x	x
Tronsco and Breads (2021)	x		x	x
Total	21	5	22	21

### 5.1 Leadership

Leadership, although not explicitly identified, was a key feature of virtually all the papers reviewed. This indicates that the way nursing leadership is communicated in the DH literature may be contributing to its relative invisibility, warranting greater consideration. A lack of explicit differentiation between nursing and midwifery was also apparent. This may be because these professional disciplines are not consistently delineated across different countries. Furthermore, being subsumed under the heading of healthcare professionals in key policy documents such as Topol (2019) exacerbates the relative invisibility of the nursing contribution to the digital agenda. Although the term e‐leadership was first used in the 1990s, we found only tacit assumptions of a shared understanding of leadership in a digital context, which remains poorly defined in the literature. Although the specific need for digital competence was emphasized, the lack of evidence regarding the suitability of transformational leadership for nurses leading digital policy and practice needs to be addressed. A new digital leadership framework (Laukka et al., 2022) provides an opportunity to do this. However, this new framework is not healthcare specific therefore its application in a DH setting, specifically in supporting nursing leadership in this context, needs to be tested.

While this review acknowledges diversity in DH adoption between countries, common challenges were faced within the global nursing community and the need to develop digital healthcare leaders and global standards that span different levels of digital maturity was apparent. Furthermore, the key role of digital nurse leaders in bringing together DH nursing specialisms (NI and telehealth) while bridging the clinical/technological divide is a priority.

The review identified nurses' involvement in planning and designing DH software and systems as a key enabler for effective practice and person‐centred care. Although we found examples of this at national/international levels, these were relatively rare, almost exclusively in developed countries, and reportedly unsustainable without a more effective and inclusive supporting infrastructure. However, the recent ICN Position Statement (ICN, 2023) offers a policy directive emphasizing the strategic role of nurses in DH which should go some way in driving the change needed.

Although leadership is required at every level (West et al., 2014), without a seat at policy and Board tables, nurses are inadequately represented in DH decision‐making. The literature reviewed highlights the emergence of specialist roles like CNIO and calls for this expert nurse leadership community to be expanded and developed. However, if existing executive nurse leaders themselves lack competence in DH, they will be less effective in developing a digitally skilled workforce; while many strategic leaders have leadership capabilities, they often lack the knowledge and expertise to recognize and respond to the DH landscape. Meanwhile, individual countries are developing context‐specific frameworks to support decision‐making (NHSE, 2022).

### 5.2 Education

The need for digital skills and competencies development for all nurses/nurse leaders was a key feature of the evidence reviewed, alongside calls for this to be a core element of health professional and clinical education (Burgess & Honey, 2022). The review findings indicated that integrated digital competence remains an ill‐defined process not widely embedded in nurse education, despite the need for healthcare professionals to develop competence in working with digital technologies and data being widely acknowledged (Booth et al., 2021; Phillips & Ives, n.d.; RCN, 2018; Topol, 2019; Wachter & Making, 2016). The review finding that competency in informatics is recognized as a central component of nursing and vital in supporting the digital patient is supported by the release of core competencies for nursing education by the American Association of Colleges of Nursing (Hussey et al., 2015; Remus & Donelle, 2019); these explicitly identify informatics, social media and emergent technologies and their impact on decision‐making and quality as critical to professional practice.

However, this review emphasizes the importance of moving beyond digital competence to capability. Although these terms are often used interchangeably, capability goes beyond the technical skill of competence to include the ability to adapt to change, self‐efficacy and lifelong learning (Saghafi et al., 2022). This distinction is important in situations involving uncertainty and incomplete information (Harrison et al., 2020) which characterize the increasing complexity of nursing. As a result, education struggles to keep pace with the challenges the profession faces in a rapidly evolving digital healthcare system.

### 5.3 Service delivery

Compassionate person‐centred care represents the bedrock of nursing practice (McCaffrey & McConnell, 2015), and the review found DH is central to achieving this in facilitating greater integration and care planning (Hussey et al., 2015; ONMSD, 2022). However, we also found evidence that the increasing popularity of remote consultations, for example, may compromise nursing ideals (de Raeve et al., 2017) as behavioural cues are missed and therapeutic exchanges minimized (Grey et al., 2023). Critics of DH argue there are possible risks to patient safety, through the reduction of face‐to‐face care and poorly designed systems, which challenge professional standards (Agnew, 2022). Historic under‐funding of IT in healthcare has led to digital inequity within health systems, health services and communities (Hutchings, 2020) with associated risks to patient safety and care quality (Agnew, 2022).

Our findings confirm relatively new, but previously identified concerns for policy leaders and practitioners about the risk of data security and patient confidentiality breaches and cyber intimidation (WHO, 2019). Risks to patient and staff safety should therefore be priority issues, aligned with DH policy and process (ICN, 2023; WHO, 2021c). Global health policy (ICN, 2023; NHS England, 2021; WHO, 2019, 2021c) acknowledges these issues, advocating that digital clinical safety should become integrated into healthcare culture, and digital solutions should be developed to improve patient and staff safety. For example, in addition to risks associated with malicious cyber‐attacks paralysing health systems or the inadvertent sharing of large personal datasets, gender‐based cyber‐threats also raise questions of intersecting inequalities in nursing (Troncoso & Breads, 2021), where 90% of the workforce is female (WHO, 2020).

The opportunities the review identified that DH creates for nurses to improve evidence‐based care. Examples include using big data science and ‘live’ patient data to inform clinical decision‐making, creating practice‐based evidence and directly improving health outcomes in alignment with the WHO Strategic Development Goals (SDGs; UN, 2015), for example, by supporting people with healthy living and exerting personal agency in systems that allow citizens to input into their DH records (WHO Europe, 2022). Our findings do not support the previously reported general disengagement of nurses from DH development but do shed light on the barriers they face. These include interoperability issues and disrupted nursing workflow, which are well‐known (RCN, 2018), as are proven solutions (Oemig & Blobel, 2022), which need to be addressed to optimize the benefits of DH. The review findings highlight the need for enhanced infrastructure to enable nursing leadership such as universal adoption of tools like the ICNP to support interoperability and improve healthcare provision. A recent position statement (ICN, 2023) echoes this, observing that international terminology standards can improve patient safety and increase stakeholder collaboration while strengthening the DH ecosystem.

### 5.4 Jobs

At a time when nursing faces its biggest global challenge in workforce planning, with a projected shortage of 5.7 million nurses by 2030 (WHO, 2020), successful digitalization of healthcare will alter the paradigm of care delivery, with potentially positive impact on workforce challenges (Velez‐Lapao et al., 2019). The COVID‐19 pandemic demonstrated that these methods could enable efficacious service provision, building capacity and skills in nursing (Dubé et al., 2020) while improving collaboration and providing an ‘enabling environment’ (WHO, 2021b). This review observed the benefits of, for example, remote consultations, telemedicine and mobile health technologies which could reduce the need for home visits or face‐to‐face appointments, free up nursing time and enhance workflow. Indeed, the ‘Building better together’ strategy around jobs (WHO, 2021d) recommends improving data collection and analysis capacity to inform staff mobilization, which the findings of our review support.

Improving health outcomes and population health naturally reduces the burden on health systems, directly impacting nursing capacity. This review identified multiple ways in which nurses can enable citizens to self‐manage, monitor health, use wearable monitoring devices to detect adverse events and access advice on illness, wellness and disease management (de Raeve et al., 2017; Honey & Westbrooke, 2016). Adopting such technology can enable nurses to access immediate results, or check for disease markers, allowing timely diagnosis and enhancing health outcomes. This means new roles for nurses as partners and health navigators, all of which align well with established nursing theory and philosophy of holism and person centredness.

The review found evidence of not only the development of digital nursing roles in several countries but also a repeat of the lack of recognition, development opportunities and career pathways previously experienced by other nurses in new roles. Similarly, the challenges posed by digitization and associated new roles such as the need to clarify professional boundaries and enabling nurses to practice within the full scope of their licence and context must be addressed by nursing leadership organizations such as National Nursing Associations and regulatory bodies. Only 5 of the 24 papers reviewed addressed the ‘Jobs’ domain of the GSDNM (WHO, 2021b), indicating a clear gap in the current evidence base that needs to be addressed because workforce availability and retention are the precursors to achievement in the other three domains.

### 5.5 Strengths and limitations

This was a robust and transparent, theory‐based review; the first of its kind on this topic. We did not, however, undertake the planned synthesis without meta‐analysis (SwiM) (Campbell et al., 2020) because none of the papers meeting the criteria for inclusion were intervention studies with quantitative outcome measures. Neither could we analyse the material by type of professional registration because this information was not reported. A strength of the review is that the integrative review methodology enabled the inclusion of a wide variety of literature. Furthermore, risk of bias was assessed using the appropriate, quality appraisal tool for each paper with the results summarized in Section 3.5 and full details provided in File S1. As indicated in Section 4 and File S1, risk of bias was present in 11 of the 24 papers reviewed. This is not uncommon in a review of this type which includes grey literature. Potential biases in the review process were minimized by publishing and adhering to the protocol, systematic application of a robust review methodology as detailed in the Methods section, in‐process critical reflection by the review team and oversight from the study steering group.

## 6 CONCLUSION

Despite the importance of DH in supporting the achievement of the UN SDGs (UN, 2015), the lack of focus on the role of nurses and midwives in the policy‐making and strategic development of DH as a key means of meeting the SDGs was evident in the WHO Europe DH Action Plan 2021–2025 (WHO Europe, 2022). This review helps to address this gap by offering a comprehensive synthesis of the evidence regarding the current contribution of nurses and midwives to leading DH policy and practice. The findings demonstrate the benefits that enabling greater nursing/midwifery leadership of DH policy and practice offers for improving health outcomes and service delivery. To release this potential, however, further research is needed to address the lack of understanding regarding what leadership is in a DH context; and to develop and test interventions to enable leadership of DH policy and practice development by nurses and midwives. This must be coupled with establishing an effective infrastructure to support the widespread, strategic nursing and leadership contribution to DH policy and practice if the promised benefits of digital transformation for enabling universal health coverage are to be realized.

## FUNDING INFORMATION

This work was supported by grants from the Royal College of Surgeons in Ireland, the Faculty of Nursing and Midwifery and the Global Nursing Leadership Institute. Representatives from the Royal College of Surgeons in Ireland Faculty of Nursing and Midwifery provided advice and guidance as part of the project advisory board but were not involved in the conduct or reporting of the review.

## CONFLICT OF INTEREST STATEMENT

The authors have no competing interests to declare.

### OPEN RESEARCH BADGES

This article has earned an Open Data badge for making publicly available the digitally‐shareable data necessary to reproduce the reported results. The data is available at [https://www.crd.york.ac.uk/prospero/#searchadvanced ID: CRD42023420369].

### PEER REVIEW

The peer review history for this article is available at https://www.webofscience.com/api/gateway/wos/peer‐review/10.1111/jan.16265.

## Supporting information


Data S1.

File S1.

File S2.

## Data Availability

The data that support the findings of this study are available from the corresponding author upon reasonable request.
