# Supplementary material for: Current nursing and midwifery contribution to leading digital health policy and practice: An integrative review
Source: J Adv Nurs. 2024 Jun 30;81(1):116–39. doi: 10.1111/jan.16265 (PMC11638528; doi:10.1111/jan.16265)
Supplement: Supplementary file 3 — File S2. [file JAN-81-116-s002.docx]

**Supplementary file 2: Theme density map**

| **Theme**  **Source** | **Theme 1:**  DH and the nursing response | | **Theme 2:**  Leadership | | **Theme 3:**  Enabling processes & tools | | **Theme 4:**  A digitally capable workforce | |
| --- | --- | --- | --- | --- | --- | --- | --- | --- |
|  | Opportunities & new ways of working | Barriers to engagement | Strategic leadership, leadership types & leader characteristics | Collaboration and bridging the clinical/technological divide | Terminology & technology | Consistent standards & guiding frameworks | Competence | Capacity |
| Agnew | x | x |  | x | x | x | x |  |
| Ahonen | x | x | x |  | x | x | x |  |
| Ariosto | x | x |  |  | x | x | x | x |
| Bakker | x | x |  | x | x | x |  |  |
| Bartz | x | x | x | x | x | x | x | x |
| Brommeyer | x | x | x | x | x | x | x | x |
| Burgess | x | x | x | x | x |  | x | x |
| De Raeve | x | x |  |  | x | x | x | x |
| Honey | x | x | x |  | x | x | x |  |
| Hussey 2015 | x | x | x | x | x | x | x | x |
| Hussey 2017 | x | x | x | x | x | x | x |  |
| Hussey 2021 | x | x | x |  | x | x | x | x |
| Ingebrigtsen |  | x | x | x |  | x | x | x |
| Laukka | x | x | x | x | x |  | x | x |
| ONMSD | x | x | x |  | x | x | x | x |
| Peltonen |  |  | x | x | x | x | x | x |
| Remus 2016a | x | x | x | x | x |  | x | x |
| Remus 2016b | x | x | x | x | x | x | x | x |
| Remus 2019 | x | x | x | x | x | x | x | x |
| Sadoughi | x |  |  | x |  | x |  |  |
| Shi |  |  |  | x | x |  |  |  |
| Strudwick | x | x | x | x | x | x | x | x |
| Tornvall | x | x |  |  |  |  | x | x |
| Troncoso | x | x | x | x | x | x | x | x |
